# Tracheal suspension with autogenous rib cartilage in a patient with severe tracheomalacia

**DOI:** 10.1186/s13019-019-0840-z

**Published:** 2019-01-25

**Authors:** Shuonan Xu, Jianfei Zhu, Guolong Zhao, Shudong Li

**Affiliations:** 1 Department of Thoracic Surgery, Shaanxi Provincial People’s Hospital, Xi’an, 710068 China; 2 The Third Affiliated Hospital of the School of Medicine Xi’an JiaoTong University, Xi’an, 710068 China; 3 The First Affiliated Hospital of Xi’an Medical University, Xi’an, 710077 China; 4 Northwest Women and Children Hospital, Xi’an, 710061 China

**Keywords:** Tracheomalacia, Tracheal suspension, Rib cartilage

## Abstract

**Background:**

Tracheomalacia (TM), caused by anterior mediastinal tumorectomy, most likely to deteriorate condition of patient life.

**Case presentation:**

A 63-year-old patient felt serious dyspnea diagnosis as TM caused by the recurrent cervical schwannoma. The narrowest diameter of the TM was only 0.446 cm and the length of malacic segment was 7.47 cm. Here we designed a novel tracheal suspension technique by using autogenous rib cartilage graft to treat severe TM. The obvious effect was observed that the inner diameter increased from 0.446 cm to 1.390 cm,and the airway symptom was alleviated.

**Conclusion:**

The autogenous rib cartilage graft used for suspending the malacic trachea was safe and effective.

## Background

Tracheomalacia (TM) is characterized by pathologically collapsed segment of cartilaginous rings and membranous wall of trachea-bronchi, which results in life-threatening symptoms [1]. TM is mainly associated with congenital malformation, surgery-based trauma and tumor compression [2]. The common surgical approach for treating TM was aortopexy over the past decades. Recently many scholars sought to stablize malacial trachea by using endotracheal stent placement and paratracheal biomaterial scaffold [3, 4], but there is no consensus with regard to radiographic assessment and standard therapeutic method for TM [2]. Here we introduced a novel tracheal suspension technique to treat a patient with TM.

## Case presentation

**Fig. 1:**
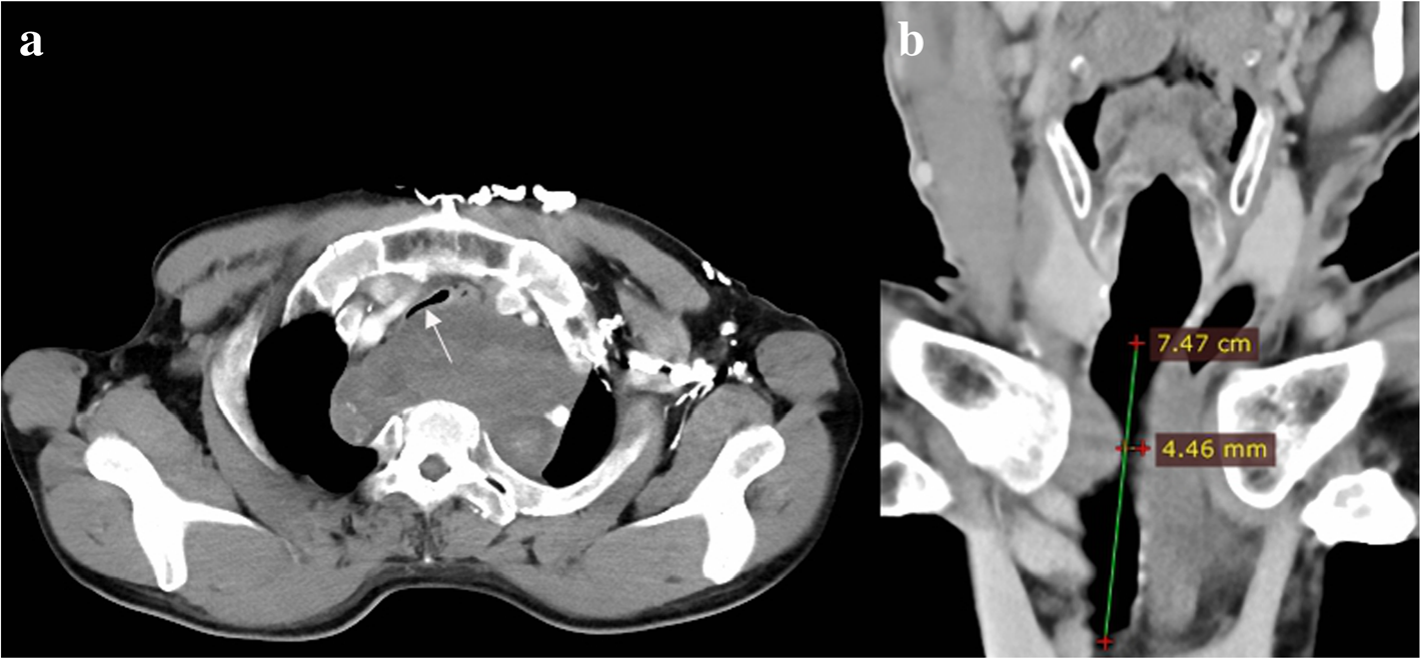
The preoperative CT image of the narrowest inner diameter of malactic trachea (**a** transverse section, **b** coronal section)

A 63-year-old male patient with large anterior mediastinal mass was referred to our hospital for treatment. The patient was pathologically diagnosed as cervical schwannoma and underwent surgical resection twelve years ago. He had re-operation because of the recurrent neck tumor four years ago. No specific neural, cardiovascular and respiratory disfunction and neoplasms his history contained as well as his family history. The patient suffered from chest oppression and shortness of breath for four months, and these symptoms gradually became worse. The Preoperative CT confirmed that the patient was diagnosed as TM and large anterior mediastinal mass (Fig. 1) Due to occasion of severe airway overreaction during the process of his endoscopy, fiber bronchoscopy was not finished.

**Fig. 2:**
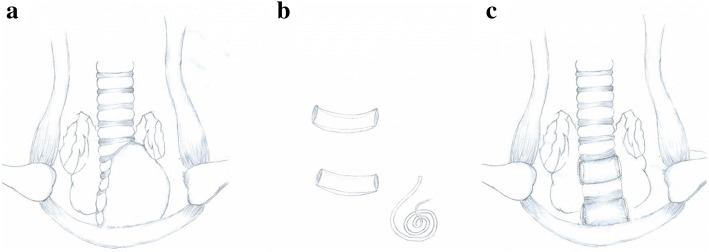
The hand drawings of prime procedures of this surgery (**a** tumor site exposure, **b** curving removed autogenous rib cartilage, **c** anchoring malacial tracheal rings and membrane by fresh graft)

Consideration of potential risk from serious TM, the patient was intubated with guidance of fibreoptic bronchoscopy in the supine position, then underwent median sternotomy and tumor resection followed by tracheal suspension. The prime procedures of this surgery were briefly depicted by hand drawings (Fig. 2a: tumor site exposure; Fig. 2b: further sculpture of removed autogenous rib cartilage; Fig. 2c: anchoring malacial tracheal rings and membrane by fresh graft) and details of surgical procedure were as follows:

Step 1: Tumorectomy. After medisection of sternum followed by opening pretracheal fascia, upper principal bronchus and frontage of cervical schwannoma were revealed. Along the line between the tumor and its adjacent tissue,the tumor was underwent entire resected.

**Fig. 3:**
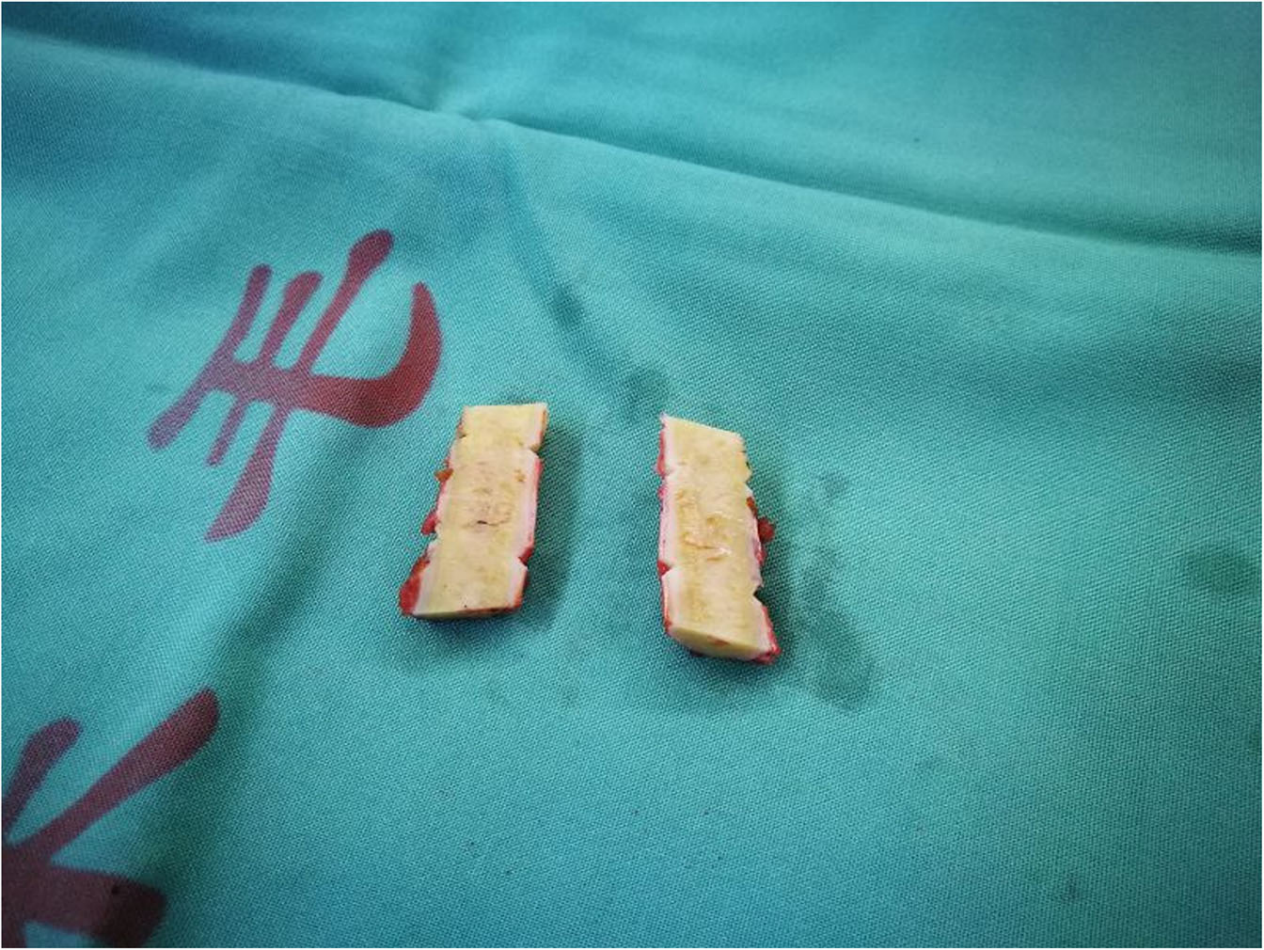
Fabrication of scaffold by using the autogenous rib cartilage

Step 2: Fabrication of scaffold. Partial autogenous rib cartilage was removed from 5th rib, and its top and bottom parts were penetrated with flexible steel needle to form two channels available for thread, which could manufactured a scaffold to anchor the extensive malacial tracheal rings and membrane (Fig. 3).

**Fig. 4:**
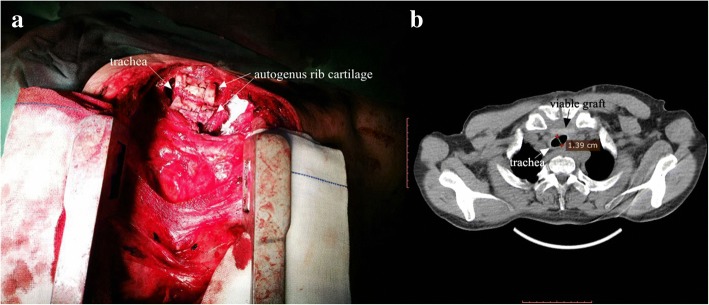
Tracheal suspension with autogenous rib cartilage (**a** intraoperative photograph of finished scaffold, **b** postoperative CT image)

Step 3: Tracheal suspension. Free rib cartilage graft, fixed with bilateral tracheal rings, were deposited in front of malacial trachea by silk thread across the channels to cover the collapsed tracheal wall, so the malactic tracheal rings and membrane were elevated and pulled for enlarging the diameter of cartilaginous ring (Fig. 4a).

After living a short period of mechanical ventilation with positive airway pressure, the patient was successfully extubated within 12 h after surgery. During his hospital stay, major postoperative complications didn’t occur, but mild pneumonia happened. The patient was discharged on the 16th day postoperatively. In the follow-up,the images showed that either cross section of intraluminal stenosis or collapsed segment of airway was remarkably relieved, and scaffold made by autogenous rib cartilage clinged to extratrachea stably. It was surprising that the graft finally integrated with tracheal wall (Fig. 4b).

## Discussion and conclusions

For most surgeons, TM-based surgeries are associated with complex approaches and lethal morbidities. Attributing to insufficient bronchotracheal blood supply, a variety of surgical procedures for TM co-existed yet none of them seemed to be the best ideal. Traditional approach for treating TM is aortopexy that surgeon make tracheal lumen be fixed with posterior aortic arch, which widely be applied to children patients [5]. However, this surgical method brings to pericardial effusion, mediastinitis, respiratory distress, relapse of disease and a high risk of death. Intraluminal stenting is the result of noninvasive persuasion, and it proved high reasonability and low postoperative complications [3, 4]. However migration of stent not only increase the rate of recurrence, also improve incidence of airway obstruction. Compared with other scaffold materials like 3D-prints and bio-syntheses, rib cartilage is easier to be obtained and higher histocompatibility, and its transplant approach is more simple as well as less cost. Takekawa et al. [6] reported two teenagers of severe chest deformity suffered from TM caused by innominate artery compression. Through autologous cartilage graft and muscle flap suspension,airway obstruction was obviously released. In our study, by utilizing the advantage of autogenous rib cartilage, we overcame the exclusive reaction and guaranteed the flow of nutrient arteries. Furthermore, the series of postoperative CT scan showed that the grafts equipped with viability creeping the cartilage rings.

Some limitations of our surgical approach could not be ignored, excessive exposing and freeing tracheal tissues result in large wound and more bleeding, also this approach has a potential risk of damaging cervical artery and essential nerve. But this method we reported is a reliable suspending technique to reduce complications for treating patient of TM with large anterior mediastinal mass. We believe that more samples of TM can be recommended to recieve this tracheal suspension.

## Abbreviations

CT: Computed tomography
TM: Tracheomalacia
